# CNVannotator: A Comprehensive Annotation Server for Copy Number Variation in the Human Genome

**DOI:** 10.1371/journal.pone.0080170

**Published:** 2013-11-14

**Authors:** Min Zhao, Zhongming Zhao

**Affiliations:** 1 Department of Biomedical Informatics, Vanderbilt University School of Medicine, Nashville, Tennessee, United States of America; 2 Department of Cancer Biology, Vanderbilt University School of Medicine, Nashville, Tennessee, United States of America; 3 Department of Psychiatry, Vanderbilt University School of Medicine, Nashville, Tennessee, United States of America; 4 Center for Quantitative Sciences, Vanderbilt University Medical Center, Nashville, Tennessee, United States of America; Huazhong University of Science and Technology, China

## Abstract

Copy number variation (CNV) is one of the most prevalent genetic variations in the genome, leading to an abnormal number of copies of moderate to large genomic regions. High-throughput technologies such as next-generation sequencing often identify thousands of CNVs involved in biological or pathological processes. Despite the growing demand to filter and classify CNVs by factors such as frequency in population, biological features, and function, surprisingly, no online web server for CNV annotations has been made available to the research community. Here, we present CNVannotator, a web server that accepts an input set of human genomic positions in a user-friendly tabular format. CNVannotator can perform genomic overlaps of the input coordinates using various functional features, including a list of the reported 356,817 common CNVs, 181,261 disease CNVs, as well as, 140,342 SNPs from genome-wide association studies. In addition, CNVannotator incorporates 2,211,468 genomic features, including ENCODE regulatory elements, cytoband, segmental duplication, genome fragile site, pseudogene, promoter, enhancer, CpG island, and methylation site. For cancer research community users, CNVannotator can apply various filters to retrieve a subgroup of CNVs pinpointed in hundreds of tumor suppressor genes and oncogenes. In total, 5,277,234 unique genomic coordinates with functional features are available to generate an output in a plain text format that is free to download. In summary, we provide a comprehensive web resource for human CNVs. The annotated results along with the server can be accessed at http://bioinfo.mc.vanderbilt.edu/CNVannotator/.

## Introduction

All human individuals are different from each other in a postulated 0.1% of genomic DNA sequences [1]. These genomic differences range from single nucleotide variants (SNVs) to large scale genomic structural variants (SVs) [2]. Recently, copy number variations (CNVs) have been discovered as a major cause of intermediate-scale structural variants in human genomes [3]. These copy number changes often refer to the alterations of DNA fragments and are involved in approximately 12% of the genome in human populations [4]. As a result of abundant CNVs in both healthy [5], [6] and diseased individuals [7], [8], CNVs introduce huge genetic variation on genes' dosage and their expression levels.

Generally, CNVs are comprised of the insertion, deletion, and duplication of DNA fragments with lengths ranging from one kilobase to five megabases [3]. Recent studies have shown that CNVs are extensively related to diseases such as cancer and neuropsychiatric disorders [7], [8], [9]. The disease-associated CNVs are typically classified into two models: rare and common CNVs [2]. Rare CNVs in the population are reportedly related to various disorders, including birth defects [10], neurological disorders [11], and predisposition to cancer [12], [13], [14]. Common CNVs collectively contribute to some complex diseases, such as HIV [15], malaria [16], chronic obstructive pulmonary disease [17], and Crohn's disease [17]. Due to their impact on human disease, CNVs can be used in both the diagnosis and treatment of diseases [18].

Cytogenetic technologies were first used to identify CNVs, such as karyotyping and fluorescence *in situ* hybridization (FISH) [19]. Later, array-based genome-wide detection of CNVs was achieved by utilizing comparative genomic hybridization (CGH) and single-nucleotide polymorphism (SNP) arrays [20]. Recently, the rapid evolution of high-throughput genotyping and next generation sequencing technologies have generated unprecedented volumes of CNV data, which provide significant study potential for a large number of genomic structure variants, including disease associated CNVs [21] and somatic CNVs leading to drug resistance in cancer treatment [22]. In recent years, the importance of accurate and unbiased annotation of CNVs has become apparent. While plenty of computational tools have been developed to detect CNVs for various platforms [21], there is still a serious informatics challenge for screening and interpreting the detected CNVs and their implicated phenotypes. To date, only two public platforms (CNV-WebStore [23] and CNV-Workshop [24]) provide limited functions to store and visualize CNVs. Therefore, there is a strong demand for comprehensive data mining across the full genomic spectrum of CNVs.

To meet this challenge, we have designed and implemented CNVannotator as a comprehensive and user-friendly web-based sever for the annotation of known CNVs and the discovery of novel CNVs. CNVannotator provides an integrative framework to interpret CNV data. The main advantage of CNVannotator is its ability to identify novel CNVs by filtering out known common and disease CNVs and other Genome-wide association studies (GWAS)-reported variants. CNVannotator also has a powerful capacity to overlap various genomic features, including gene fusion sites, segmental duplication sites, genomic fragile sites, cytoband, and pseudogene. In addition, it is useful for the annotation of regulatory elements, including promoter, enhancer, CpG island, methylation site, and microRNA target regions. Since a large number of CNVs are implicated to be associated with cancers [8], we provided cancer-specific annotation features according to thousands of cancer mutations and hundreds of known coding and non-coding tumor suppressors and oncogenes.

## Materials and Methods

**Figure 1:**
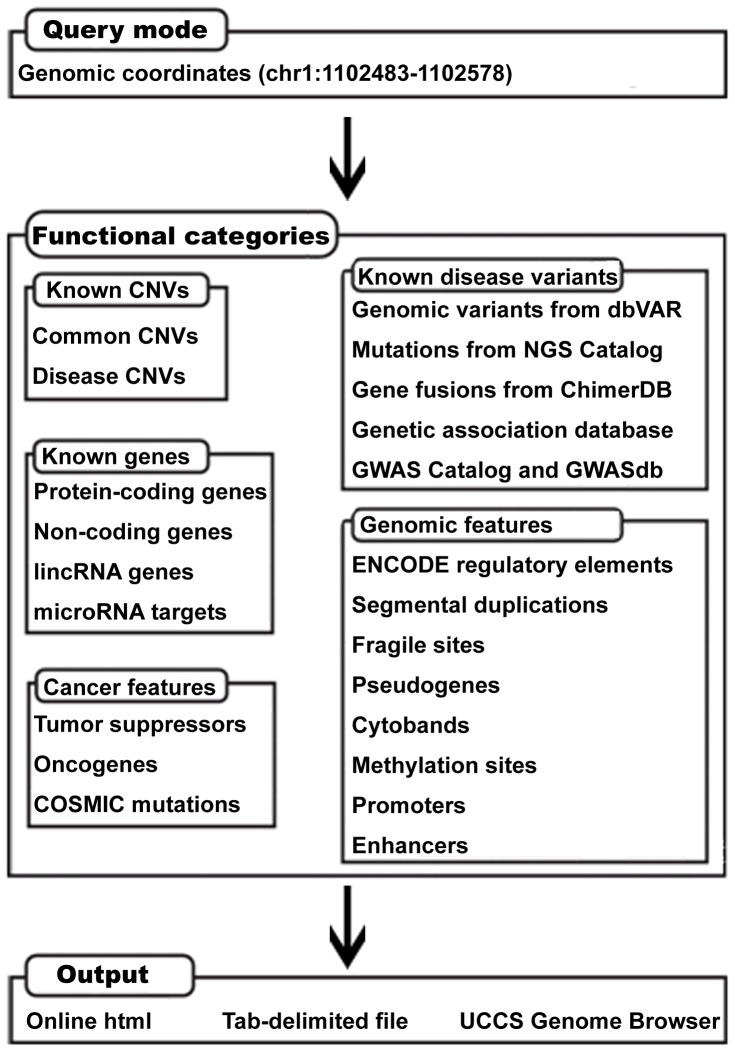
The input, annotation categories, and output of CNVannotator.

In this section, we describe the integration of various biological databases to annotate CNVs. We imported 24 genomic and functional annotations, including known CNVs, known variants related to disease, protein-coding and non-coding genes, genomic features, and cancer-specific features, from 18 databases (Table 1). In total, 5,277,234 unique genomic coordinates with functional or genomic features are seamlessly integrated into CNVannotator to produce a downloadable annotation output in a plain text format (Figure 1). More annotation data will be added to CNVannotator as it becomes available.

**Table 1:** All the annotations in CNVannotator web server.

Data source	Number of genomic coordinate	Source and reference
**Known CNVs**		
Common CNVs	356,817	Common CNVs from DGV database [25]
Disease CNVs	181,261	Disease CNVs from CNVD database [26]
		
**Known variants**		
dbVar	2,716,881	Genomic structural variants in dbVAR [27]
GWASdb	137,111	Human genetic variants by GWAS [31]
GWAS Catalog	6381	Etiologic and functional variants [30]
GAD	3057	Genetic variants by association studies [32]
Gene fusion	1198/1103^a^	Experimentally validated gene fusion events from ChimerDB [29]
NGS Catalog	1071	Genetic variants from NGS-based studies in human [28]
**Coding and non-coding genes**		
microRNA target	52,920	Targeting gene for all human miRNAs [35]
Coding gene	30,770	Protein-coding RefSeq genes [34]
Long non-coding RNA	21,033	Long non-coding genes (UCSC browser [34])
Other non-coding RNA	1337	Non-coding genes from UCSC browser (Excluding long non-coding RNAs) [34]
**Genomic features**		
ENCODE regulomeDB	1,880,556	Genomic functional elements from ENCODE data [37]
Segmental duplication	40,832	Global analysis result of human segmental duplications [38]
Promoter	29,119	500 bp upstream from the transcription start sites using UCSC data [34]
CpG island	28,691	CpG island data from UCSC browser [34]
Methylation	19,754	Human disease methylation sites from DiseaseMeth database [40]
Pseudogene	11,983	Pseudogene data from UCSC browser [34]
Enhancer	1478	Enhancer data from UCSC browser [34]
Cytoband	862	Cytoband data from UCSC browser [34]
Fragile site	69	Human genomic fragile sites from Entrez gene database [39]
**Cancer genomic features**		
COSMIC	125,753	Somatic mutations in cancer [41]
Tumor suppressor	716	Coding and non-coding tumor suppressor genes from TSGene database [42]
Oncogene	263	Coding oncogenes integrated from UniProt and TAG databases [43]

a Two numbers represent the unique genomic regions for the fusion gene pairs.

### Common and disease CNV data collection

The primary purpose of this study is to provide a CNV list to filter and annotate experimental results for large scale genomic CNV studies. For SNV annotation, researchers often exclude common SNPs and focus on phenotype-specific rare variants. Similar to this SNV annotation approach, common CNVs can be filtered out to narrow the focus toward phenotype-specific CNVs. To this purpose, we retrieved 356,817 common CNVs stored in the Database of Genomic Variants [25]. To assist phenotype-specific CNV annotation, 181,261 disease-related CNVs were integrated from The Copy Number Variation in Disease [26]. By combining a large number of known common and disease CNVs, researchers can systematically filter and classify the CNVs of interest based on population and disease information. In addition, users can query relevant CNVs for any interesting genes based on our collection.

### Known genomic variants data collection

To help researchers overlap CNVs with other genomic variation events, we compiled 2,716,881 genomic SVs from dbVAR [27], which consists of genomic deletion, insertion, inversion and other complex structural alterations. Additionally, 1246 more genomic variants with phenotype information and 777 non-redundant gene fusion events were integrated from the NGS Catalog database [28] and ChimerDB 2.0 [29], respectively. In terms of known SNVs, we integrated 140,342 SNPs that are associated with diseases or phenotypes in GWAS from the NHGRI GWAS Catalog [30] and GWASdb [31] along with 40,136 disease-related records from the Genetic Association Database (GAD) [32]. Overlapping these compiled SNVs and SVs from several datasets with differing quality criteria might lead to the identification of recurrent genomic abbreviation events.

### Gene annotation collection

To assist with mapping CNVs to gene function, we integrated 30,770 protein-coding genes from the RefSeq database [33] and 939 microRNAs, 402 small nucleolar RNAs (snoRNAs), and 21,033 long non-coding genes from UCSC Genome Browser [34]. In addition, 52,920 microRNA targeting genes were integrated from TargetScan [35]. This comprehensive collection of coding and non-coding genes may help researchers to address how many genes exist in variable numbers of copies, which provide clues for further gene dosage-relevant exploration.

### Genomic features collection

CNVs represent the copy number change of a DNA fragment in the genome. The genomics environment may provide clues to explain the formation and function of CNVs. For instance, segmental duplication near ancestral duplication sites may increase the probability of regional duplication, which may result in CNVs [36]. To help researchers explore the genomic features of CNVs, 1,902,632 functional sites from the ENCODE project that affect the transcription factor, DNase binding, and eQTL features were integrated from the RegulomeDB database [37]. Furthermore, 40,832 segmental duplication events were integrated from the Segmental Duplication database [38]. Using a keyword search, we retrieved 69 fragile chromosome sites from the Entrez Gene database [39]. Additionally, 19,754 methylation sites for various human diseases were integrated from the DiseaseMeth database [40]. Moreover, CNVannotator incorporated 72,133 genomic features from UCSC Genome Browser [34], including pseudogene, promoter, enhancer, CpG island, and cytoband information.

### Cancer-specific annotation collection

CNVannotator provides three cancer-specific annotation types, including cancer somatic mutations, tumor suppressor genes, and oncogenes. Specifically, CNVannotator can apply filters to retrieve 125,753 cancer mutations in the COSMIC database (Version 65) [41]. For the important cancer genes, we integrated 716 human tumor suppressor genes from the Tumor Suppressor Gene Database (TSGene) [42]. In addition, we integrated a gene set of 296 protein-coding oncogenes that included oncogenes if they were supported by both the UniProtKB keyword “Proto-oncogene” [43] and the Tumor Associated Gene (TAG) database (http://www.binfo.ncku.edu.tw/TAG/).

## Results and Discussion

All data and annotation information in CNVannotator are stored in a MySQL-based database on a Linux server (Figure 1). In this section, we first represent two main views of search results (gene-based and genomic region-based query) in CNVannotator. Next, we present the results of a specific application of CNVannotator in which it annotates the top 10 novel CNVs from microsatellite stable hereditary nonpolyposis colorectal cancer (MSS HNPCC) [44].

### Gene-based view to retrieve CNV information

**Figure 2:**
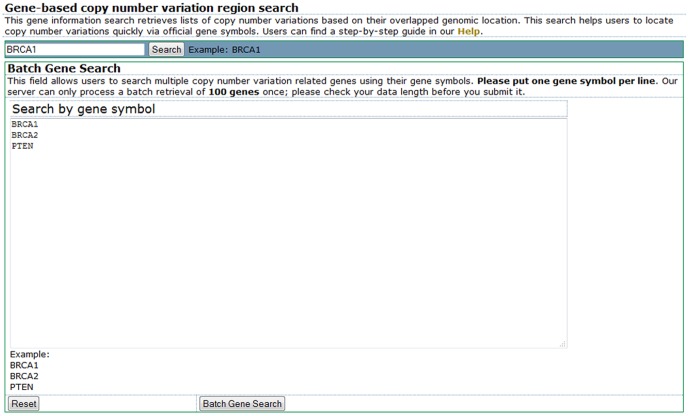
The layout of CNVannotator gene-based query viewer. The single gene and multiple gene query interfaces are shown. Both queries require an input of the official gene symbol(s).

All the common and disease CNVs and their annotations in our database are searchable. The gene-based view can help researchers retrieve a list of CNVs for their genes of interest (Figure 2). We provided two types of query interfaces for single genes or multiple genes. Using a gene symbol-based keyword search, researchers can quickly obtain information on the number of CNVs stored in our CNVannotator server related to the searched gene. For a list of genes, we provided a batch retrieval interface, through which researchers can search CNVannotator using a list of human gene symbols. Gene data exactly matching the input gene symbols are used to retrieve their related CNVs. In the input page, users are required to input their gene symbols line by line. The search results in a list of overlapped CNVs and a hyperlink to access the original references for the reported CNVs.

### Region-based view to annotate CNVs

**Figure 3:**
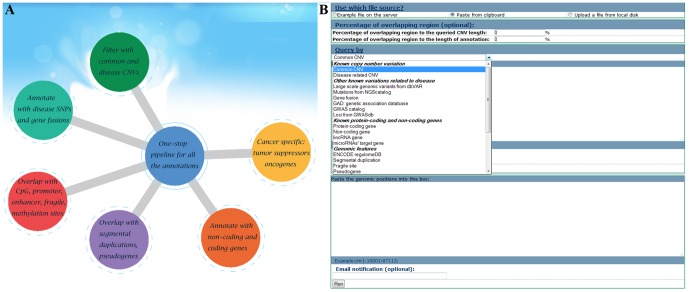
The genomic region-based query viewer in CNVannotator. (A) The access to various analytic tools for numerous functional annotation modules. (B) The web interface to input a list of CNVs for annotation. In the drop-down menu, users can choose the most relevant annotation option.

The genomic region-based query is used to annotate CNVs according to genomic coordinate information (Figure 3). From the CNVannotator homepage, users can quickly access web interfaces for a variety of annotations (Figure 3A). In this section, the detailed information regarding input options and results in CNVannotator are presented one by one.

#### CNVannotator input options

A precise overlap of a user's input information to the relevant genomic annotation in the CNVannotator database is a critical foundation for follow-up data interpretation. For the genomic region-based query, it accepts a set of genomic positions in a tabular format (e.g., starting and ending coordinates in each chromosome). Users can either paste their input genomic positions into the online form or upload a text file containing all the required information for CNV annotation (Figure 3B). Additionally, a user may provide their email address (optional) on the input page. Upon submission of a user's query, an email containing the computational job information is sent to the user.

To provide more flexible options to pinpoint overlapping regions, users can set the percentage of overlapping regions to both the queried CNV length and the genomic length of annotation (Figure 3B). Only the annotations that are equal to or higher than the two percentages will be returned. The two parameters are optional, and the default values are zero. By applying strict overlapping percentages, only features that are highly similar to the input will be returned. On the other hand, users can obtain more annotations by lowering the overlapping percentages. However, for the annotations associated with a single base pair, the overlapping percentage parameters are not applicable. In our implemented CNVannotator web server, there are four single nucleotide-based annotations, including SNVs from GWASdb, gwasCatalog, GAD, and COSMIC. For these four annotations, we only implemented the direct overlapping function for the input CNV coordinates.

#### One-stop mode and detail query mode

One of the basic functions of our implemented overlapping program scans chromosome regions from the user's input (genomic location) against all the biological annotations with those genomic coordinates. For instance, the user's input may include a set of detected CNVs, and the goal is to find out whether the inputted regions have been implicated in previously reported, closely-related diseases. In another scenario, the user may intend to filter out the common CNV in large populations. For each annotation in Table 1, we provide the specific overlapping algorithm to retrieve the researcher's desired information. To facilitate more efficient annotation of CNVs, we provided not only the access to annotations with 24 different features in our database, but also a one-stop result retrieval interface to annotate all the information (Figure S1). In the one-stop mode, the researchers can annotate all of the 24 features for any interesting CNV regions.

The results from CNVannotator fall into four main classifications. Firstly, the common CNVs provide users with an overview of the CNVs in normal populations. Secondly, the disease CNVs and other disease-related variations, including SNVs and SVs, help to prioritize related disease information associated with inputted CNVs. Thirdly, the segmental duplication regions and fragile sites may assist the user to understand whether the CNVs are located in chromosomal rearrangement hotspots. Finally, regulatory information, including regulatory elements from the ENCODE data, are useful to clarify the regulatory mechanism that are biological significant.

#### The result file and job retrieving system

**Figure 4:**
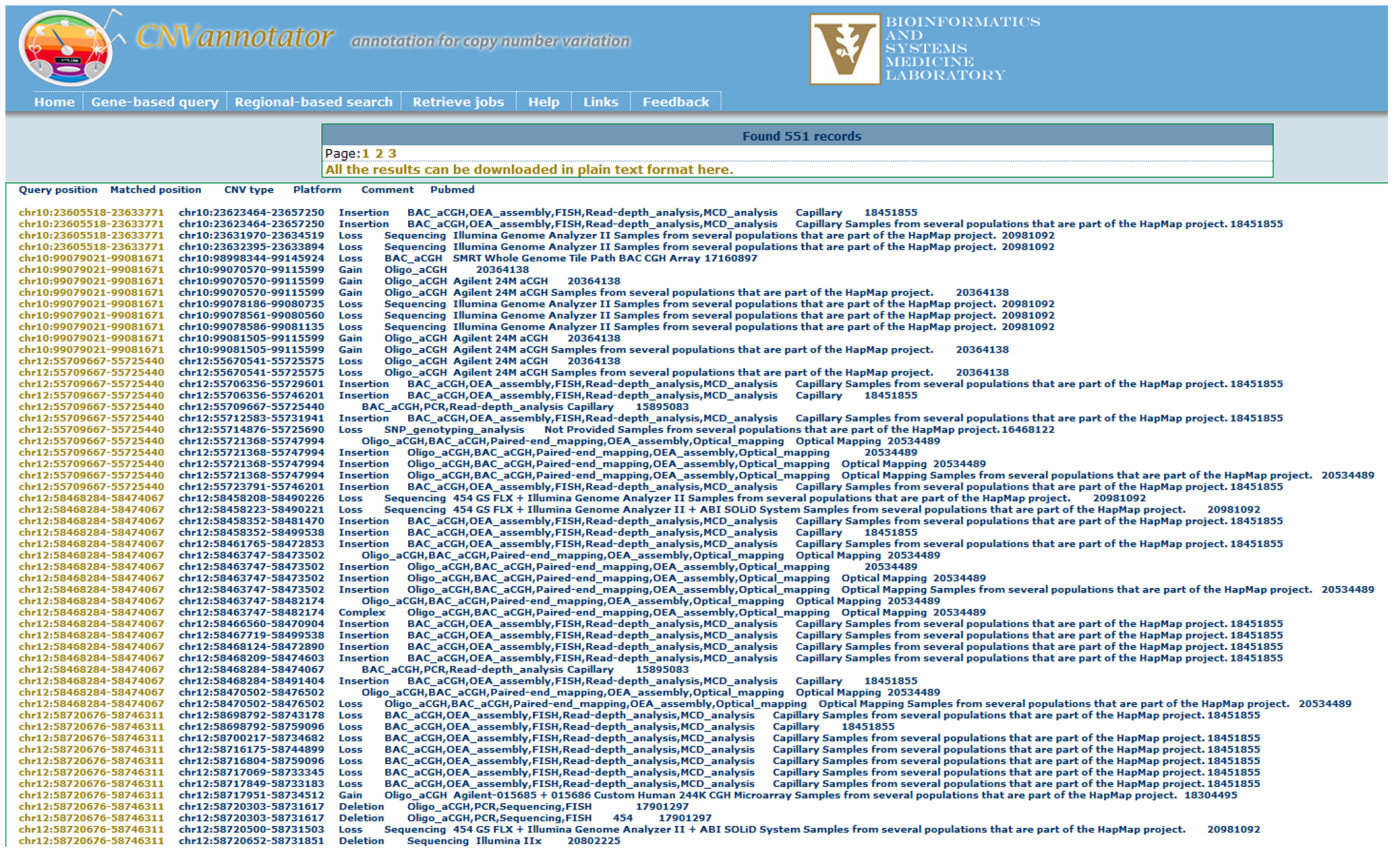
An example of the CNVannotator region-based search result layout. After successfully uploading a CNV list(s), a set of annotations are overlapped to the input CNVs and are represented in by hyperlinks to the UCSC Genome Browser. Additionally, the tabular text file is available to download for further filtering and classification.

Dynamic CNV results that match the genomic coordinates in each query will be displayed on the user's end (e.g., web browser). For the matched genomics regions, the UCSC Genome Browser hyperlinks are provided as an opportunity to explore more genomic features (Figure 4). In addition, a downloadable text file is provided for researcher to manipulate or filter data using external tools (e.g., Excel, SPSS, SAS, R/S-Plus, and others).

All completed jobs submitted to CNVannotator are saved in the server for two weeks. The job retrieval system was implemented for both single and multiple jobs (Figure S2). By inputting the job ID generated from CNVannotator, researchers can download their finished jobs promptly in a text file format. For a list of jobs, CNVannotator provides a batch retrieval mode. This procedure first requires uploading a job ID list containing any number of job identifiers generated by the CNVannotator web server. The hyperlinks to the matched jobs will be displayed in the web browser for instant download.

### Annotation for cancer CNVs

CNVannotator aims to systematically extract biological meaning from CNV lists derived from high-throughput genomic experiments. To demonstrate the usefulness of this server, we annotated 10 recently reported novel CNVs from microsatellite stable hereditary nonpolyposis colorectal cancer (MSS HNPCC) [44]. The aim of the genome-wide study was to identify the potential genomic regions relevant to MSS HNPCC. Using CNVannotator's default setting (i.e., the overlapping region percentage for both the queried CNV length and the genomic length of annotation are zero, which returns any overlapping regions between input and annotations in CNVannotator), ten CNVs were overlapped with 141 common CNVs and 1432 disease CNV events (Table 2). Interestingly, the ten CNVs were also mapped to 88 cancer mutations, 3 tumor suppressor genes, and 2 oncogenes. Among the 1432 disease CNV records, 1081 were reported from various cancer samples (Table S1). In addition, we identified 104 gene fusion events, 28 microRNA target genes, 19 methylation sites, 17 segmental duplication regions, 16 promoters, 6 long non-coding RNAs, and 3 genome fragile sites in the top ten novel CNVs in MSS HNPCC. These features may provide new insights into the complex genetic changes associated with MSS HNPCC. For example, the 19 disease-related methylation sites overlap with the promoter regions of seven genes: *CAPZA2*, *PCCA*, *RAPGEF5*, *ST7*, *USP43*, *ZIC2*, and *ZIC5*. Interestingly, the expression of *RAPGEF5* exhibited as an altered protein expression in the other 41 independent MSS HNPCC samples [44].

**Table 2:** The annotation results for the top ten novel CNVs from microsatellite stable hereditary nonpolyposis colorectal cancer samples using the CNVannotator web server.

Data source	Number of annotations
Structure variants from dbVAR	2008
Disease CNVs	1432
Common CNVs	141
Gene fusion events	104
Cancer mutations	88
Significant SNPs from GWASdb	82
The microRNA target genes	28
Known protein-coding genes	27
Methylation sites in promoter region	19
Segmental duplication regions	17
Promoters regions	16
Cytobands	10
CpG islands	8
Long non-coding RNAs	6
Pseudogenes	4
Tumor suppressor genes	3
Fragile sites	3
Significant SNPs from GWAS catalog	2
Oncogenes	2

### Future direction

High-throughput technologies often result in large genomic regions, such as CNVs, that are related to the biological condition in investigation. Our web resource CNVannotator performs a complete CNV annotation pipeline and provides easy access to intermediate and final results through a user-friendly interface. The advantages of this platform's features include: ability to roughly explore CNVs one by one; rapid assessment of whether expected/important genes are in the CNV list; ability to display and save all annotations in a linear tabular text format for other external analyses; provides links to genome browser for more information around the given annotation; and, well-suited for the analysis of a small number of focused CNVs. Due to genomic structure complexity, the analysis of CNVs is more likely an exploratory, computational procedure instead of a purely statistical framework. Typically, the related CNVs scattered in the result may lose interrelationships during exploration. It is still challenging to differentiate between important CNVs or non-specific CNVs. We may improve the web server when more data [45] and a practical statistical frameworks [46], [47] become available.

### Conclusion

Data analysis of CNVs is an important downstream task to mine the biological meaning of resultant CNV lists from large scale genomic experiments. The analysis of such high complexity and large volume data sets is challenging and requires support from specialized bioinformatics software packages. We have implemented an interactive web server, CNVannotator, for an in-depth analysis of CNVs based on information from common and disease CNVs, genomic structure variants, inferred physical segmental duplication and fragile sites, reported disease phenotypes, annotated regulatory elements, as well as specific information about the cancer genomics. The current version of CNVannotator comprehensively collected 5,277,234 unique genomic coordinates with functional or genomic features from 18 databases. CNVannotator was designed for multiple purposes, including (i) overlapping known common CNVs with disease CNVs, (ii) annotating CNVs that have structure variation events, (iii) overlapping millions of genomic features to explore the regulatory or functional elements in human genome, and (iv) automating the annotation for cancer-relevant mutations and important genes.

CNVannotator was specifically developed to help minimize the manual work incurred when annotating a list of CNVs using a set of biological/genomic data sources. This platform provides considerable capabilities for researchers to annotate specific CNVs in a reliable and efficient manner. Further development of the program will be focused on updating data regularly as new versions become available, including additional genomic/functional annotation, as well as improving the interactive web interface.

## Supporting Information

Figure S1 **The one-stop model for region-base query in CNVannotator.**
(TIF)

Figure S2 **The job retrieval system in CNVannotator.** (A) Finished job access resulting from inputting single or multiple job IDs. (B) Job retrieval results, which include hyperlinks for the downloadable tabular result files.
(TIF)

Table S1 **The annotation results for the top ten novel CNVs from microsatellite stable hereditary nonpolyposis colorectal cancer samples using CNVannotator web server.**
(XLSX)
