# Supplementary figures and images for: CNVannotator: A Comprehensive Annotation Server for Copy Number Variation in the Human Genome
Source: PLoS One. 2013 Nov 14;8(11):e80170. doi: 10.1371/journal.pone.0080170 (PMC3828214; doi:10.1371/journal.pone.0080170)

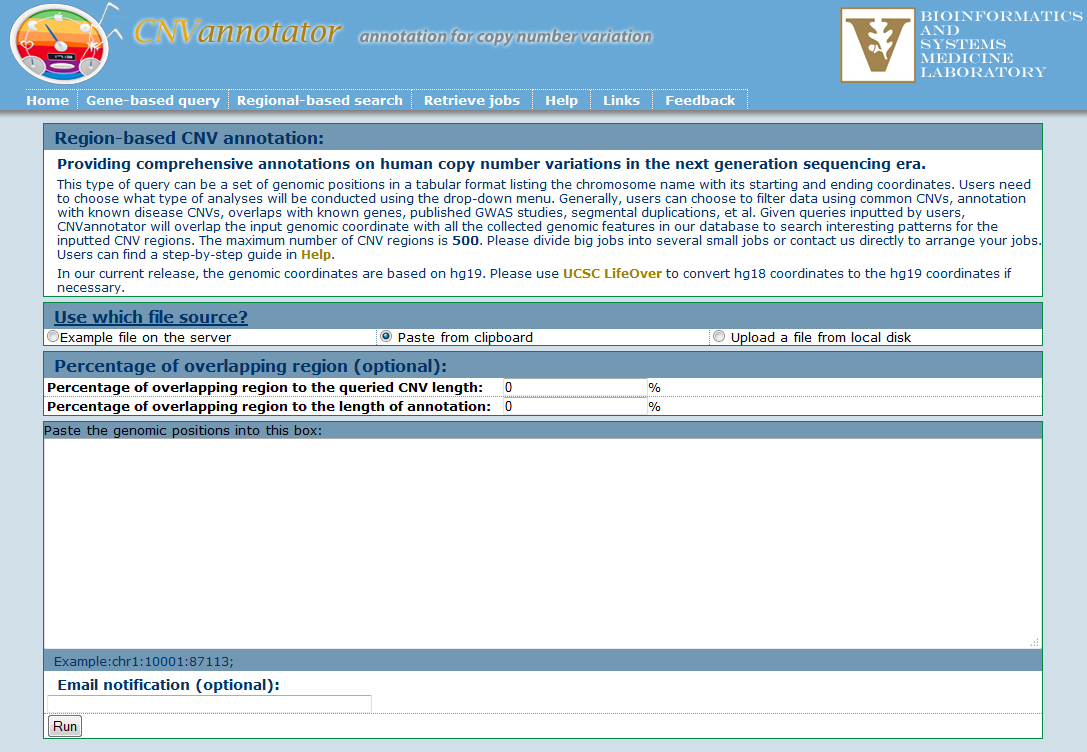

Supplement: Figure S1 — The one-stop model for region-base query in CNVannotator. (TIF) [file pone.0080170.s001.tif]

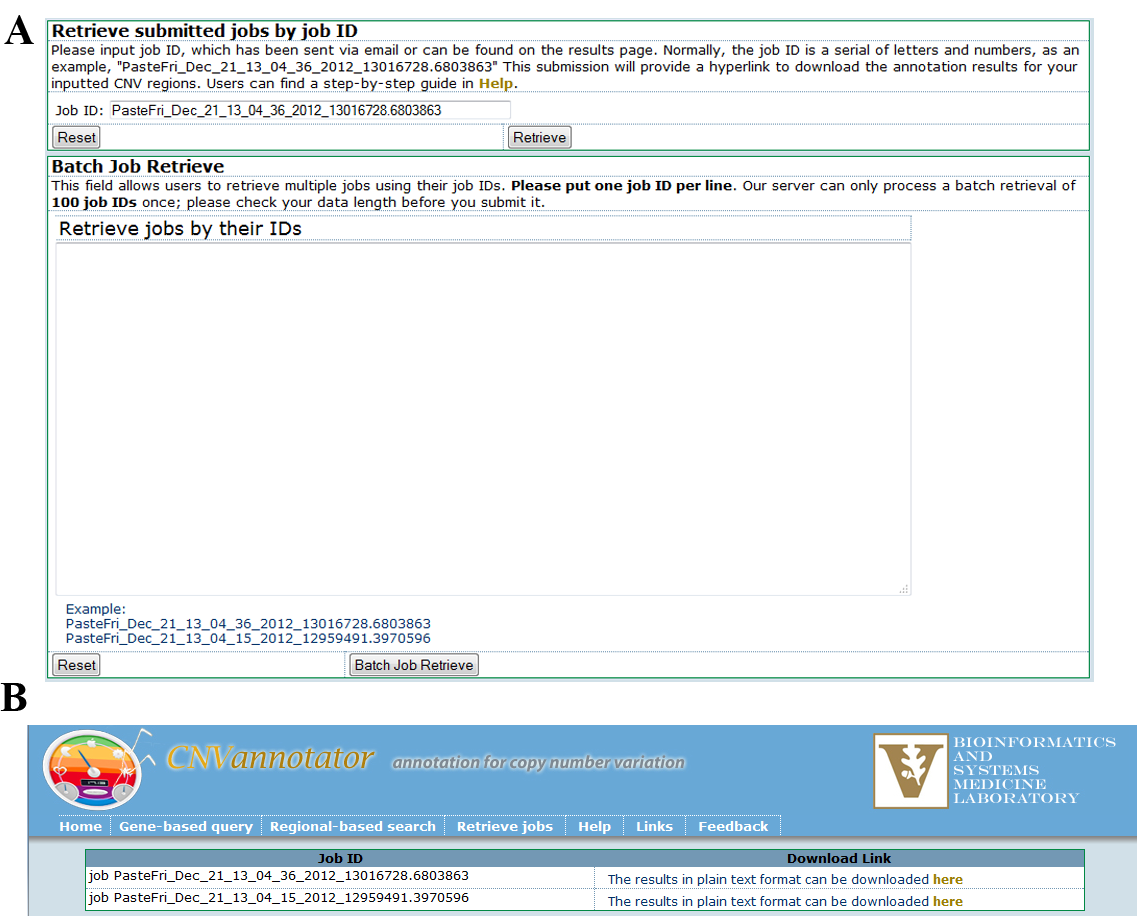

Supplement: Figure S2 — The job retrieval system in CNVannotator. (A) Finished job access resulting from inputting single or multiple job IDs. (B) Job retrieval results, which include hyperlinks for the downloadable tabular result files. (TIF) [file pone.0080170.s002.tif]
